# A techno-psychological approach to understanding problematic use of short-form video applications: The role of flow

**DOI:** 10.3389/fpsyg.2022.971589

**Published:** 2022-08-17

**Authors:** Qing Huang, Mingxin Hu, Ning Zhang

**Affiliations:** ^1^College of Media and International Culture, Public Diplomacy and Strategic Communication Research Center, Zhejiang University, Hangzhou, China; ^2^School of Public Health, Second Affiliated Hospital of Zhejiang University School of Medicine, Hangzhou, China

**Keywords:** problematic SVA use, technological affordances, recommendation algorithm, multimodality, low-cost interaction, flow

## Abstract

Short-form video applications (SVAs) have been gaining increasing popularity among users, which has raised the concern of problematic SVA use. Flow—a positive experience in which individuals feel immersion, enjoyment, temporal dissociation, and curiosity—contributes to the development of problematic SVA use. Most of the prior research examined the motivations of flow and the self-traits that trigger flow, but paid limited attention to the technological affordances of smartphone applications that facilitate users' flow. Algorithm recommendation, multimodality, and low-cost interaction are three affordances of SVAs. Thus, drawing upon the stimulus-organism-response (S-O-R) framework, this study proposes a mediation model to examine how these affordances influence problematic SVA use through flow. An online survey (*N* = 621) showed that algorithm recommendation was negatively associated with problematic SVA use but was not significantly correlated to flow. Multimodality was directly and positively associated with problematic SVA use. Meanwhile, the relationship between these two variables were mediated by flow. Low-cost interaction had an indirect link with problematic SVA use via flow, while the direct link between them was not significant. The results suggest that low-cost interaction is the affordance that is most likely to trigger flow and problematic SVA use, followed by multimodality. However, algorithm recommendation seems to be an affordance that is less likely to facilitate flow or cause problematic SVA use. Our proposed model not only enriches the S-O-R framework in the digital environment, but also denotes a techno-psychological approach to examine problematic use of SVAs and other digital applications. Moreover, the findings offer practical implications for optimizing SVAs' technological affordances to properly manage problematic SVA use.

## Introduction

If you have ever used short-form video applications (SVAs, e.g., TikTok), you may have such a feeling that “a perceived minute spent on SVAs actually takes several hours in life” (Guangming Daily, 2021). The setting of full-screen auto-play and the customized recommendations of vivid contents afford users immersive experiences when using SVAs (Zhang et al., 2019; Zhao, 2021). These user experiences made SVAs increasingly popular in recent years. By the end of 2021, the number of SVA users in China has reached 934 million, witnessing an increase of 60.8 million users compared with 2020 (China Internet Network Information Center, 2021). Moreover, TikTok, a popular SVA worldwide, has received more than a billion active monthly users by the third quarter of 2021 across the globe (Daily Economic News, 2021).

However, overuse of SVAs tends to result in negative consequences. In the year of 2021, users across China spent approximately 2 h on SVAs on average each day (China Network Audiovisual Program Service Association, 2021). Moreover, news media frequently reported how excessive SVA use impaired heavy users' physical health and psychological wellbeing. For instance, during the addictive use of SVAs, teenagers tended to imitate characters in the short-form videos, which rendered them vulnerable to the undesirable or maladaptive behaviors performed by some characters (People's Daily, 2022). Besides, spending too much time on SVAs distracted young adults from work, reducing work efficiency or exacerbating procrastination (Guangming Daily, 2022). Additionally, a growing number of seniors have become increasingly glued to SVAs, and their unlimited use has impaired eyesight, caused cervical spine deformity, and triggered family disputes (China Daily, 2021).

Scholars have termed the above-mentioned phenomena as problematic SVA use, which describes an individual's inability to control SVA use and the associated outcomes (Wang et al., 2021; Huang et al., 2022a). Drawing on prior research (Zhang et al., 2019; Liu et al., 2021; Wang et al., 2021; Huang et al., 2022a), problematic SVA use consists of four dimensions: (1) loss of control, namely, engaging in compulsive SVA usage for uncontrolled time duration; (2) withdrawal, that is, experiencing unpleasant feelings with suspension of SVA use; (3) craving, that is, going through mood changes if SVAs are not available; and (4) negative life consequences, referring to the physical and psychological impairment due to excessive SVA use.

Considering that SVAs are a newly developed web-based application installed on smartphones, research on problematic smartphone use and problematic Internet use offers insights into understanding problematic SVA use. Prior studies mainly adopted the psychopathological approach and the compensatory use approach to examine users' problematic use of various web-based technologies. The psychopathological approach assumes that individuals with predisposed psychopathologies, such as depression, social anxiety, and substance dependence, are vulnerable to maladaptive cognitions and social isolation, which leads to problematic SVA or Internet use behaviors (Davis, 2001; Elhai and Contractor, 2018; Elhai et al., 2020). By contrast, the compensatory use approach is developed from the uses and gratifications theory and sees individuals as active actors (Elhai et al., 2017). This approach posits that individuals use online applications to regulate their negative emotions, while the addiction-like symptoms resulting from overuse are unintended side effects (Kardefelt-Winther, 2014; Wegmann and Brand, 2019).

Despite the different assumptions about the nature of users, the psychopathological approach and the compensatory use approach have one thing in common: the positive feelings generated from using the web-based technologies could either help users with psychopathological conditions regain meaning or compensate for users' stressful life situations. These positive feelings, resulting from the reward mechanism of the human brain, are the key to developing problematic use behaviors (Koob and Le Moal, 2008). Notably, flow—an optimal experience in which an individual is fully immersed in an activity due to a feeling of energized focus, full involvement, enjoyment, and success in the process of the activity (Csikszentmihalyi, 1990)—is the key indicator of the reward mechanism that explains people's problematic use of smartphones and various online applications (Thatcher et al., 2008; Khang et al., 2013; Mazzoni et al., 2017; Wang, 2020; Barberis et al., 2021; Brailovskaia et al., 2021). Nevertheless, most of these studies examined the motivations of flow or the self-traits that are highly likely to trigger flow (Khang et al., 2013; Mazzoni et al., 2017; Wang, 2020; Barberis et al., 2021), whereas paid limited attention to the technological characteristics of the web-based applications that facilitate users' flow.

Given that the technological features of SVAs play an important role in the development of problematic SVA use (Zhang et al., 2019), this study focuses on the technological antecedents of flow. Drawing on the stimulus-organism-response framework (Fang et al., 2017, 2021; Tuncer, 2021), we examine how three technological affordances of SVAs, namely, recommendation algorithm, multimodality, and low-cost interaction afford users' experience of flow, which in turn, contribute to their problematic use of SVAs. By testing the mediating role of flow between three affordances and problematic SVA use, we propose a techno-psychological approach to understand the problematic use of SVAs. Such an approach not only helps us differentiate between how certain technological affordances induce problematic SVA use *via* flow, but also offers insights into effectively managing problematic SVA use through optimizing SVAs.

## Literature review and hypotheses development

### The stimulus-organism-response framework

The stimulus-organism-response (S-O-R) theory is used as the overarching framework of the current study. Developed from behavioral psychology, the S-O-R framework maintains that clues perceived from an environment (S) triggers an individual's internal assessment (O), which subsequently activates this person's behavioral response (R) (Mehrabia and Russell, 1974). In recent years, the S-O-R framework has been widely used to explicate people's digital media use behaviors (Fang et al., 2017, 2021; Tuncer, 2021). For instance, the perceived affordances of online commerce platforms (e.g., visibility, interactivity, selectivity) influenced users' assessment of the brand experience and the relationship quality with the seller, which in turn, regulated their purchase intention and further engagement with the platform (Fang et al., 2021; Tuncer, 2021). Likewise, users' perception of mobile travel applications' design and performance affected their psychological engagement and benefit perception of the applications, which then drove their behavioral engagement with mobile travel applications (Fang et al., 2017). Moreover, the technological features of SVAs—such as immersion, socialization, and control—induce addictive usage by activating users' perceived enjoyment and feeling of withdrawal (Tian et al., 2022).

To sum up, the above-reviewed studies updated the S-O-R framework in the digital environment: affordances of digital media perceived by users (S) affect their assessment of the media and the associated psychological experiences (O), which in turn, influence their digitally mediated behaviors (R). According to this updated S-O-R framework, we try to explain how certain technological affordances of SVAs (S) trigger a user's flow experience (O), and thereby induce the user's problematic SVA use (R).

### Technological affordances and problematic SVA use

The concept of affordance was first coined by Gibson (1979) in the field of ecological psychology. Originally, Gibson defined an affordance as a possibility for action available in the environment, which exists as congenial to the action capabilities of an organism (Gibson, 1979). Such a definition highlights that an affordance manifests a certain relationship between an organism and its surrounding environment. Later, to modify Gibson's model of affordance, Norman (1988) accommodated design interests and introduced a perspective of subjective perception to understand affordance: an affordance is a perceived property of a thing that determines how the thing could possibly be used by a human being (Norman, 1988, 1990). Norman (1988) developed Gibson (1979) definition in two ways. First, only when an affordance is perceived by an actor, instead of being an objective possibility for action, can the affordance induce a behavior. Second, focusing on affordances perceived by human actors rather than organisms in general helps explicate how to optimize the design of a thing for human beings' better usage. Notably, the increasing digitalization in the past decades has drawn researchers' interest from affordances of things to affordances of technologies. The term technological affordance has thus frequently appeared in recent scholarship (Leonardi, 2011; Majchrzak et al., 2013; Lu et al., 2014; Chatterjee et al., 2015; Abhari et al., 2017). Accordingly, based on Norman (1988, 1990) approach to affordance and draws on research on affordances of information technologies (Leonardi, 2011; Majchrzak et al., 2013; Lu et al., 2014; Chatterjee et al., 2015; Abhari et al., 2017), this study defines technological affordances as the features of a digital technology that may invite certain behaviors recognized by users.

Given that contents, forms, and the mode of human-computer interaction constitute a typical scenario of technology use, we draw upon prior research (Sundar and Limperos, 2013; Zhang et al., 2019; Zhao, 2021) and propose three affordances of SVAs that induce users to consume contents (i.e., recommendation algorithm), utilize various forms to edit (i.e., multimodality), and interact with the interface in specific ways (i.e., low-cost interaction). Recommendation algorithm is widely used in the design of various mobile applications, through which the platforms tailor information and services according to users' preferences, thereby increasing user satisfaction (Moon et al., 2014; Mou et al., 2021). In reference to previous research (Zhang et al., 2019), we consider recommendation algorithm a content-related affordance and define it as the perceived potential of SVA platforms to provide users with customized contents and services based on their preferences. The use of certain modalities—the ways in which information is presented, such as text, audio, and audiovisual—has a great impact on how the information is received and processed (Sundar and Limperos, 2013). Given the design of SVAs, this study sees multimodality as a form-related affordance and defines it as users' perceptions of SVAs' possibility of presenting information in multiple forms through the function of filters, frames, templates, and beautifying. Besides, users have frequently reported that SVAs are quite easy and convenient to use, in that they just need to continuously slide up the screen to view the auto-play videos (Zhao, 2021). This delineates the low-cost interaction affordance (Chen et al., 2017), an affordance that characterizes the human-computer interaction and allows SVA users to watch short-form videos with the least cognitive effort yet obtaining instant gratifications.

Affordances of a given technology tend to invite users to interact with the technology in certain ways to reach their goals (Norman, 1988, 1990; Withagen et al., 2012). For instance, the technological affordances of TikTok influence users' experience, thereby affecting their intention to use TikTok for health information seeking (Song et al., 2021). Likewise, the technological affordances of social networking sites contribute to users' satisfactory use, thus increasing their stickiness to these sites (Shao et al., 2020). Besides, in the context of organization management, affordances of social media applications facilitate business organizations' communicative practices with stakeholders, thereby improving the organization-stakeholder relationship (Argyris and Monu, 2015). These studies suggest that technological affordances have the potential to elicit a certain mode of behavior among users. Moreover, considering that recommendation algorithm, multimodality, and low-cost interaction represent an affordance that is related to contents, forms, and human-computer interaction modes, respectively, we try to explore whether these affordances of SVAs may induce the problematic use behavior in different ways. Specifically, we ask the following research question:

**RQ1**: Is recommendation algorithm (RQ1.1), multimodality (RQ1.2), and low-cost interaction (RQ1.3) significantly associated with problematic SVA use, respectively? Are there any differences in the associations between three affordances and problematic SVA use?

### Technological affordances and flow

Flow represents an immersive and optimal experience in which an individual gets fully absorbed in an activity and feels heightened enjoyment, curiosity, and little distinction between self and the environment (Csikszentmihalyi, 1990). According to the S-O-R framework in the digital environment (Fang et al., 2017, 2021; Tuncer, 2021), it is the setting and design of a technology that induces users' experience with the technology (Ghazali et al., 2018). Likewise, we assume that the technological affordances of SVAs are the antecedents that trigger users' flow experience.

Recommendation algorithm affords SVA users customized contents and services based on their preferences. For instance, if you viewed several videos about food, the algorithm would push food-related videos when you use SVAs next time. The frequent exposure to the tailored contents makes it easier for users to concentrate and reach the state of cognitive absorption (Huang, 2016). This is because contents tailored to an individual's interest create few cognitive obstacles for this person to get fully involved. Moreover, a plethora of research has demonstrated that one's cognitive absorption in a limited field of stimuli largely facilitates this person to experience flow (Chou and Ting, 2003; Lee et al., 2017). Short-form videos recommended by algorithms represent an array of limited and concentrated stimuli that are highly likely to trigger viewers' cognitive absorption. Thus, we infer that recommendation algorithm of SVAs may elicit users' flow:

**H1**: Recommendation algorithm is positively associated with flow.

Multimodality of SVAs enables users to receive and process short-form videos in multiple forms with varying special effects. For example, the filters, frames, and templates of SVAs provide different scenarios to present a video, such as scenery spots and movie-like settings. Likewise, the beautifying function of SVAs allows characters to modify or improve their outlook in a video. These various modalities enhance the richness of the experience and arouse users' multiple senses (Coyle and Thorson, 2001; Jung and Sundar, 2018), thereby facilitating them to experience sensory immersion (Shin, 2019), as if they were there in the scenario presented by the short-form video. Moreover, studies have demonstrated that multiple modalities of digital technology create a sense of being there, thus providing users with an uninterrupted and immersive experience of flow (Huang, 2016; Kim and Ko, 2019). In a similar vein, we expect that multimodality of SVAs creates a sensory rich environment and enriches users' senses. When immersed in such an environment, users tend to experience flow. Thus, we posit the following hypothesis:

**H2**: Multimodality is positively associated with flow.

Low-cost interaction of SVAs reduces the effort to watch short-form videos to a minimal level and provides users with instant gratifications (Zhao, 2021). For instance, users just need to slide up the smartphone screen to view the auto-played short-form videos, from which they get relaxed and have fun immediately. In most circumstances, the convenience and ease of using a technology increase users' psychological engagement with this technology, whereas complexity of use may prevent a user from deeply engaging with the technology (Peters et al., 2016). In relation to this study, when users recognize that SVAs are easy to use and require little effort to operate, they tend to be psychologically engaged with these applications. A high level of psychological engagement indicates a state of full involvement, thus inducing an experience of flow. Meanwhile, obtaining short-term and prompt rewards enables users to focus on the essentials of an activity, thereby creating an immersive feeling in using a technology (Przybylski et al., 2010; Suh et al., 2017). Accordingly, instant gratifications acquired during users' interaction with SVAs are highly likely to trigger their flow experience. Given that the low-cost interaction affordance is characterized by convenience, ease of use, and instant gratifications (Chen et al., 2017), we put forward the following hypothesis:

**H3**: Low-cost interaction is positively associated with flow.

### Flow and problematic SVA use

Flow is an important concept developed in positive psychology (Csikszentmihalyi, 2014). It represents an optimal experience that one is fully involved in an activity. Flow is characterized by the feeling of concentration, enjoyment, curiosity, and intrinsic interest (Csikszentmihalyi and LeFevre, 1989). In addition to the field of positive psychology, flow has been widely examined in the context of information technologies and has been used as a powerful conceptual tool to understand problematic use of information technologies (Chou and Ting, 2003; Kim and Davis, 2009; Duke and Montag, 2017).

The brain's reward mechanism helps explain the relationship between flow and problematic SVA use. Problematic and addictive behaviors are rooted in the neurobiological model of the brain's emotional system (Koob and Le Moal, 2008). Specifically, when the brain receives external stimuli such as gambling, playing games, and drugs, the secretion of dopamine increases, which allows an individual to obtain a reward, such as monetary benefits and psychological satisfaction. Meanwhile, the positive feeling of getting a reward further reinforces the individual to perform the same behavior over and over again. Noticeably, this reward mechanism not only compensates for the reward deficit in the short term, but also inhibits the anti-reward system in the long run, which results in problematic and addictive behaviors (Volkow et al., 2002; Gardner, 2011).

In the context of SVA usage, users who experience flow may perceive the state of concentration, enjoyment, and transcendence of self as an intrinsic reward. The continuous reward then facilitates users to spend more time on SVAs and engage deeper in short-form videos, which finally leads to problematic use of SVAs. Indeed, prior research has demonstrated that flow generated from using online applications tends to facilitate users to develop problematic use behaviors (Wu et al., 2013; Wang, 2020). Therefore, we propose that people who experience flow during SVA usage are more likely to engage in problematic SVA use. Accordingly, we posit the following hypothesis:

**H4**: Flow is positively associated with problematic SVA use.

### The mediating role of flow

The positive associations between three affordances and flow and the association between flow and problematic SVA use suggest a mediation model, in which flow mediates the relationship between three affordances and problematic SVA use, respectively. The mediating paths are in line with the S-O-R framework (Mehrabia and Russell, 1974; Fang et al., 2021; Tuncer, 2021), which assumes that certain affordances of a digital technology (e.g., recommendation algorithm, multimodality, and low-cost interaction) influence users' experiences in using this technology (e.g., flow), which in turn affects users' subsequent behaviors (e.g., problematic SVA use). Thus, we posit a set of hypotheses to test the mediating role of flow between three affordances and problematic SVA use:

**H5**: Flow mediates the relationship between recommendation algorithm (H5.1), multimodality (H5.2), low-cost interaction (H5.3) and problematic SVA use, respectively.

Moreover, given that recommendation algorithm, multimodality, and low-cost interaction demonstrate the possibilities of using SVAs in different ways, users' flow afforded by each of the three affordances may differ. Hence, we ask the following research question:

**RQ2**: Are there any differences in the indirect effects described in H5?

**Figure 1:**
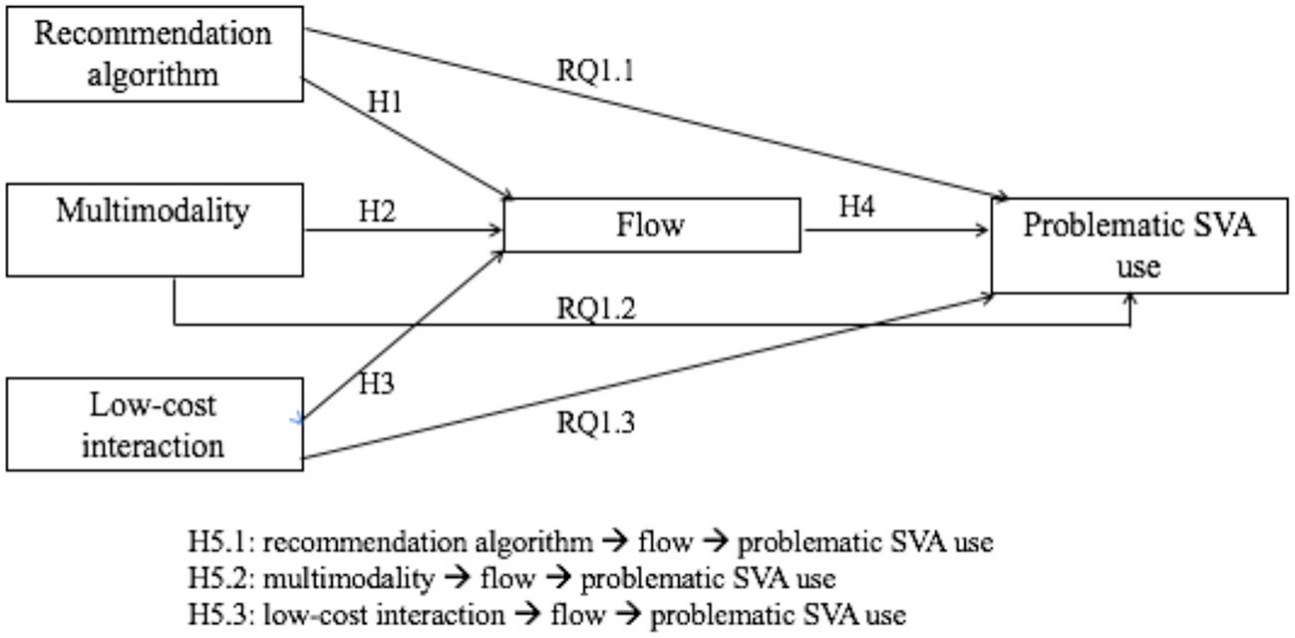
The hypothesized model.

Figure 1 presents the hypothesized model of this study.

## Materials and methods

### Participants

We collected data using Sojump's sampling service. Sojump is a professional data collection platform in China and provides a sampling pool consisting of around 2.6 million participants nationwide. This sampling strategy has been frequently used in studies that examined users' online behaviors (Cui and Wu, 2021; Huang et al., 2022b; Rui, 2022; Zhang and Fan, 2022). The survey was conducted from 14 March 2022 to 30 March 2022. In terms of sampling procedures, each registered user in the Sojump survey pool has an ID number in the range from 1 to 2,600,000. The company automatically generated random integers between this range, obtaining a sample size as requested by the researchers. The company then sent an email to invite the selected users to participate in the online survey. Questionnaires were accessible *via* mobile phones, tabloids, and personal computers. The data collection protocol was approved by the institutional review board of the authors' affiliated university. Voluntary informed consent was obtained from the participants before the online survey.

To be eligible for our study, participants should have the experience of using SVAs. Meanwhile, cases were considered invalid if they met one of the two criteria: (1) submitted the questionnaire multiple times using the same IP address; and (2) failed any of the 4 attention checks (e.g., please select “strongly agree”). Finally, we collected 621 valid questionnaires for data analysis. Table 1 presents the demographic characteristics of our participants.

**Table 1:** Demographic characteristics of the participants.

**Measure**	**Item**	**Frequency**	**Percentage (%)**
Age	18–24	83	13.4
	25–34	379	61.0
	35–44	120	19.3
	45–54	33	5.3
	55–99	6	1.0
Gender	Male	326	52.5
	Female	295	47.5
Monthly income	Less than 1,500 RMB	18	2.9
	1,501–2,000 RMB	14	2.3
	2,001–3,000 RMB	21	3.4
	3,001–5,000 RMB	81	13
	5,001–8,000 RMB	205	33
	8,001–12,000 RMB	150	24.2
	12,001–20,000 RMB	111	17.9
	More than 20,000 RMB	21	3.4
Education level	Never attend to school	0	0
	Primary school	0	0
	Middle school	7	1.1
	High school	17	2.7
	Vocational high school	13	2.1
	Higher vocational school	64	10.3
	Bachelor	478	77
	Master	41	6.6
	PhD	1	0.2
	Beijing, Shanghai, Tianjin, Chongqing, Shenzhen	200	32.2
	Capital city of province	245	39.5
Area	Prefecture-level cities	145	23.3
	Counties and towns	30	4.8
	Administrative villages	1	0.2

### Measures

#### Recommendation algorithm

Referring to prior studies on the personalized recommendation algorithm of SVAs (Li et al., 2021; Song et al., 2021), this study compiled a five-item instrument to measure recommendation algorithm. Sample items included: “SVAs can provide me with personalized videos tailored to my everyday life” and “SVAs push contents that suit my interest”. Items were measured on a five-point scale (1 = “strongly disagree”, 5 = “strongly agree”) and averaged to create a composite index (*M* = 4.22, *SD* = 0.47, Cronbach's α = 0.70).

#### Multimodality

We developed a scale of multimodality based on the special effects afforded by TikTok, such as filters, frames, templates, beautifying, and voice changing (Liu, 2022). Multimodality was measured with three items on a five-point scale (1 = “strongly disagree”, 5 = “strongly agree”): (1) SVAs offer various special effects, such as filters, frames, templates, and beautifying, which make video editing interesting, (2) SVAs offer various special effects, which make video watching attractive; and (3) SVAs offer various special effects, which increase my willingness of continuous SVA usage. Items were averaged, with higher scores indicating stronger perceptions of the multimodality affordance (*M* = 3.96, *SD* = 0.75, Cronbach's α = 0.80).

#### Low-cost interaction

According to the prior measurement of low-cost interaction of SVAs and smartphones (Yoon and Kim, 2007; Chen et al., 2017; Zhao, 2021), we created an eleven-item scale with three dimensions, namely, (1) convenience (e.g., “Using SVAs is effortless for me”), (2) ease of use (e.g., “When using SVAs, I just need to continuously slide up to view the videos”) and (3) instant gratifications (e.g., “I use SVAs because they fulfill my needs immediately”). Items were asked on a five-point scale (1 = “strongly disagree”, 5 = “strongly agree”). The internal consistency of the scale was acceptable (*M* = 4.15, *SD* = 0.46, Cronbach's α = 0.79). To test the construct validity of the scale, we conducted a confirmatory factor analysis. According to the acceptable thresholds of the model fit indices (Bentler, 1992), the factor structure of the newly compiled scale showed a good fit: χ^2^*/df* = 3.06, GFI = 0.96, CFI = 0.93, RMSEA = 0.06, SRMR = 0.04. Additionally, the factor loadings of all items on their respective dimensions exceeded the recommended cut-off value of 0.40 (Hassim et al., 2020).

#### Flow

Drawing upon previous research (Trevino and Webster, 1992; Agarwal and Karahanna, 2000; Wang et al., 2020), we modified the scale of flow in the context of SVA usage, which included thirteen items with four dimensions: (1) temporal dissociation (e.g., “Sometimes I lose track of time when I am using SVAs.”); (2) focused immersion (e.g., “While using SVAs, I am able to block out most other distractions.”); (3) heightened enjoyment (e.g., “I have fun interacting with SVAs.”); and (4) curiosity (e.g., “Using SVAs excites my curiosity.”). Questions were asked on a five-point scale (1 = “strongly disagree”, 5 = “strongly agree”), with higher scores suggesting high levels of flow experience (*M* = 3.97, *SD* = 0.54, Cronbach's α = 0.87).

#### Problematic SVA use

Problematic SVA use was measured using an established instrument (Huang et al., 2022a). The instrument consisted of thirteen items with four dimensions: (1) loss of control (e.g., “I find myself engaged on SVAs for a longer period of time than intended.”), (2) withdrawal (e.g., “I feel anxious if I have not checked for SVAs updates for some time.”), (3) craving (e.g., “I often think of SVAs when I am doing something else.”), and (4) negative life consequences (e.g., “Feeling pain in the wrists or at the back of the neck while using SVAs.”). The participants answered the questions on a five-point scale (1 = “strongly disagree”, 5 = “strongly agree”). The items were averaged to create a composite index, with a higher value indicating a greater tendency of engaging in problematic SVA use (*M* = 3.09, *SD* = 0.76, Cronbach's α = 0.91).

#### Control variables

We included five control variables in the hypothesized model: duration of SVA use, lack of self-control, gender, education level, and monthly income. We used a single item to measure duration of SVAs use: “On average, how long do you use SVAs every day?” Answers were scored on a ten-point scale, ranging from less than 10 min to more than 5 h (*Median* = 5.00 or 1–1.5 h, *SD* = 1.76). Adapted from the prior instrument (Tangney et al., 2004; Busch et al., 2021), three items were used to assess lack of self-control, such as “I have a hard time breaking bad habits.” Gender was measured as a dichotomous variable (52.6% males), while monthly income (*Median* = 5.00, or 5001–8000 RMB, *SD* = 1.45) and education level (*Median* = 7.00, or bachelor's degree, *SD* = 0.80) were measured as ordinal variables.

### Statistical analyses

We first used SPSS version 26.0 to calculate the means and standard deviations of the examined variables and the Pearson correlations between them. Then, we used AMOS version 23.0 to conduct a path analysis to test the research hypotheses. Technological affordances of SVAs (i.e., recommendation algorithm, multimodality, and low-cost interaction) and control variables were treated as exogenous variables. Endogenous variables included flow (i.e., the mediator variable) and problematic SVA use (i.e., the outcome variable). We tested the direct and indirect effects with 5,000 bootstrap samples at 95% confidence intervals (Preacher and Hayes, 2008). Standardized coefficients were reported.

## Result

### Preliminary analysis

Table 2 presents the means, standard deviations, and bivariate correlations between the examined variables. The mean value of problematic SVA use was 3.09 on a five-point scale, indicating a medium level of problematic SVA use among our participants. Recommendation algorithm (*r* = 0.52, *p* < 0.01), multimodality (*r* = 0.57, *p* < 0.01), and low-cost interaction (*r* = 0.68, *p* < 0.01) were all significantly and positively correlated with flow. Meanwhile, a positive correlation between flow and problematic SVA use was found (*r* = 0.57, *p* < 0.01). Additionally, recommendation algorithm (*r* = 0.24, *p* < 0.01), multimodality (*r* = 0.36, *p* < 0.01), and low-cost interaction (*r* = 0.43, *p* < 0.01) were positively linked to problematic SVA use. Among the control variables, lack of self-control (*r* = 0.47, *p* < 0.01) and duration of SVA use (*r* = 0.24, *p* < 0.01) were positively associated with problematic SVA use, suggesting that people with lower levels of self-control and a longer duration of SVA usage were more likely to engage in problematic SVA use.

**Table 2:** Means, standard deviations, and bivariate correlations between examined variables.

**Variables**	**M**	**SD**	**1**	**2**	**3**	**4**	**5**	**6**	**7**	**8**	**9**	**10**
1. Recommendation algorithm	4.21	0.47	-									
2. Multimodality	3.96	0.75	0.49**	-								
3. Low-cost interaction	4.15	0.46	0.65**	0.54**	-							
4. Flow	3.97	0.54	0.52**	0.57**	0.68**	-						
5. Problematic SVA use	3.09	0.76	0.24**	0.36**	0.43**	0.57**	-					
6. Lack of self-control	2.90	0.92	−0.02	−0.04	0.10*	0.12**	0.47**	-				
7. Gender	1.48	0.50	0.002	0.003	−0.03	−0.05	0.01	0.08*	-			
8. Education level	6.80	0.80	0.04	0.04	0.08	0.08*	0.07	−0.03	0.01	-		
9. Monthly Income	5.32	1.45	0.10*	0.14**	0.10*	0.13**	0.03	−0.14**	−0.14**	0.31**	-	
10. Duration of SVA use	5.35	1.76	0.25**	0.21**	0.25**	0.26**	0.24**	0.12**	−0.002	0.000	0.06	-

Gender 1 = male, 2 = female; ^*^ p < 0.05; ^**^ p < 0.01.

### Path analysis

**Figure 2:**
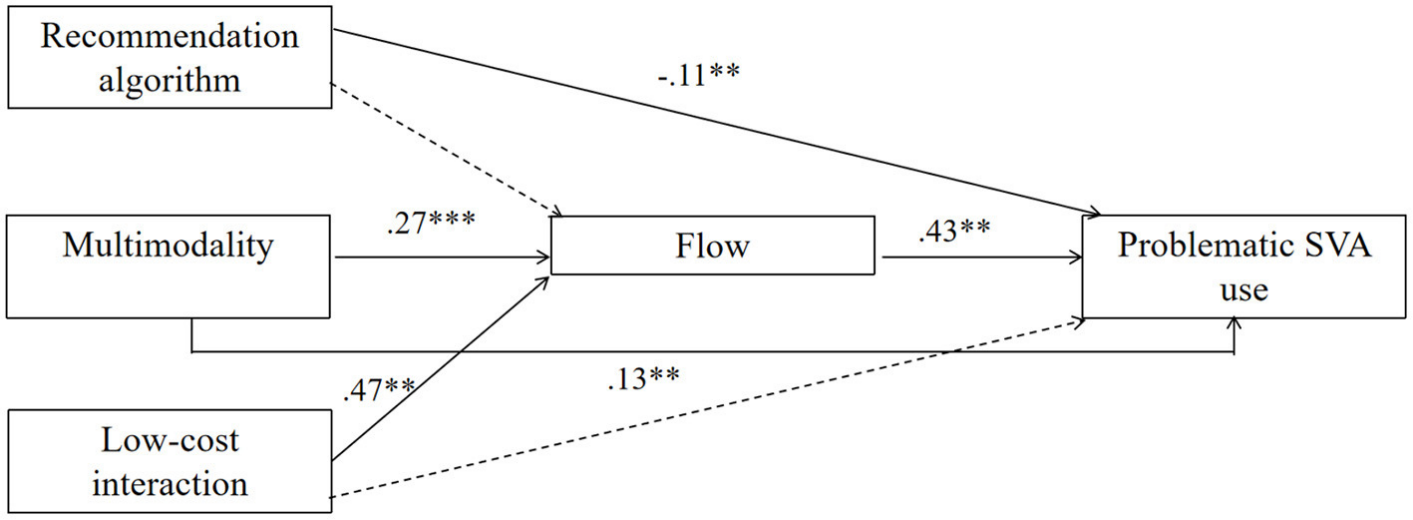
Path model diagram. ***p < 0.001, **p < 0.01, *p < 0.05.

We tested a model in which recommendation algorithm, multimodality, and low-cost interaction were included as independent variables. Flow was treated as the mediator and problematic SVA use was entered as the dependent variable. Based on the acceptable thresholds of model fit indices (Hu and Bentler, 1999; Kline, 2005)^1^, our model demonstrated a good fit: χ^2^ (6) = 7.15, *p* = 0.31, CFI = 0.99, TLI = 0.99, GFI = 0.99, RMSEA = 0.02, SRMR = 0.01. Our model explained about half of the variance in problematic SVA use (R^2^ = 0.51). Table 3 demonstrates the results of path analysis and Figure 2 presents the path model diagram.

**Table 3:** Path analysis results.

	**Independent variable**	**β**	**95% CI**	***p***
**Direct effect**				
Problematic SVA use	Recommendation algorithm	−0.11	−0.19, −0.03	<0.01
	Multimodality	0.13	0.04, 0.23	<0.01
	Low-cost interaction	0.09	−0.01, 0.19	0.08
	Flow	0.43	0.34, 0.53	<0.01
Flow	Recommendation algorithm	0.07	−0.09, 0.18	0.42
	Multimodality	0.27	0.20, 0.35	<0.001
	Low-cost interaction	0.47	0.36, 0.56	<0.01
**Indirect effect**				
Problematic SVA use	Recommendation algorithm	0.03	−0.04, 0.09	0.41
	Multimodality	0.11	0.08, 0.16	<0.01
	Low-cost interaction	0.20	0.14, 0.27	<0.01
**Total effect**				
Problematic SVA use	Recommendation algorithm	−0.08	−0.18, 0.01	0.07
	Multimodality	0.25	0.16, 0.33	<0.001
	Low-cost interaction	0.29	0.20, 0.38	<0.001

*β*, standardized regression coefficient; CI, confidence interval.

#### Direct effects

To answer **RQ1**, recommendation algorithm (β = −0.11, *p* < 0.01) and multimodality (β = 0.13, *p* < 0.01) were directly linked to problematic SVA use. However, low-cost interaction was not significantly associated with problematic SVA use (β = 0.09, *p* = 0.08).

#### Indirect effects

Inconsistent with **H1**, the relationship between recommendation algorithm and flow was not significant (β = 0.07, *p* = 0.42). Supporting **H2**, multimodality was positively correlated with flow (β = 0.27, *p* < 0.001). Likewise, the association between low-cost interaction and flow was also significant (β = 0.47, *p* < 0.01), showing support for **H3**. Besides, in line with **H4**, flow was positively associated with problematic SVA use (β = 0.43, *p* < 0.01).

To test the mediating role of flow, the results showed that the indirect effect of recommendation algorithm on problematic SVA use through flow was not significant (β = 0.03, *p* = 0.41). Thus, **H5.1** was not supported. In comparison, multimodality was indirectly linked to problematic SVA use via flow (β = 0.11, *p* < 0.01), showing support for **H5.2**. Additionally, the indirect link between low-cost interaction and problematic SVA use through flow was also significant (β = 0.20, *p* < 0.01), thereby supporting **H5.3**. To answer **RQ2**, the results demonstrated that the indirect effect of low-cost interaction on problematic SVA use was the strongest, followed by the indirect effect of multimodality, whereas the indirect effect of recommendation algorithm on problematic SVA use was not significant.

## Discussion

Flow plays an important role in developing problematic use of smartphones and online applications (Thatcher et al., 2008; Khang et al., 2013; Mazzoni et al., 2017; Wang et al., 2020; Barberis et al., 2021; Brailovskaia et al., 2021). Although previous research has identified the motivations of flow or the personality traits that tend to trigger flow (Khang et al., 2013; Mazzoni et al., 2017; Wang et al., 2020; Barberis et al., 2021), few studies have examined the technological antecedents that induce flow. To this end, the current study proposes a techno-psychological approach and uses a mediation model to test the mediating role of flow between affordances of SVAs and problematic SVA use. The results showed that flow positively mediated the association between two affordances—multimodality and low-cost interaction—and problematic SVA use. Nevertheless, due to the non-significant relationship between recommendation algorithm and flow, the mediating role of flow between recommendation algorithm and problematic use of SVAs was not significant. In terms of the direct links, recommendation algorithm was negatively linked to problematic SVA use, multimodality was positively related to problematic SVA use, whereas the direct association between low-cost interaction and problematic SVA use was not significant. Implications for understanding the relationship between each affordance and problematic SVA use are discussed.

### Theoretical implications

First, the non-significant association between recommendation algorithm and flow and the negative association between recommendation algorithm and problematic SVA use might be related to the stage of SVA use. At the early stage, users would be curious and enjoyed about the contents recommended by SVAs that were tailored to their preferences. The curiosity and enjoyment on the one hand facilitated users to experience flow (Agarwal and Karahanna, 2000), while on the other hand stimulated them to watch more videos, which finally led to problematic use. Hence, the hypothesized positive association between recommendation algorithm and flow and that between recommendation algorithm and problematic SVA use apply to explaining the early-stage users. However, at the later stage, users might get fed up with the homogenous contents recommended by algorithms. The boredom then prevented one from continuously watching videos, thereby lowering the likelihood of developing problematic use. Meanwhile, given that arousal and the desire for challenge are important prerequisites for experiencing flow (Jin, 2012), the boredom resulted from repeated exposure to homogenous contents undermined these prerequisites, thus cutting the link between recommendation algorithm and flow. Notably, on average, our participants had used SVAs for more than 2 years (Mean = 6.10, on a seven-point scale, 1= “less than a month”, 7 = “more than 3 years”), indicating that the majority of them were at a later stage of usage when this study was conducted. Accordingly, whether recommendation algorithm increases people's flow and their tendencies of engaging in problematic SVA use may depend on their stage of use.

Second, multimodality was not only indirectly associated with problematic SVA use *via* flow, but was also directly linked to problematic SVA use. The indirect association corroborated the S-O-R framework in the digital environment (Fang et al., 2017, 2021; Tuncer, 2021), in which technological affordances (i.e., multimodality) induced users' experience (i.e., flow), and thereby influenced the behavior of technology use (i.e., problematic SVA use). Besides, considering that multimodality is a form-related affordance, the direct association between multimodality and problematic SVA use has two implications. On the one hand, form-related affordances could directly affect users' behavioral patterns of SVA use, such as the problematic use. On the other hand, flow might not be the only mediating mechanism underlying the association between multimodality and problematic SVA use. In addition to flow, certain motivations may mediate the relationship between multimodality and problematic SVA use. For instance, multimodality affords users opportunities to present themselves through video shooting and kill time with the vivid contents. Meanwhile, motivations of passing time and self-presence predict people's problematic use of online applications (Khang et al., 2013; Wang et al., 2020). These findings suggest potential mediating factors underlying the relationship between multimodality and problematic SVA use.

Third, low-cost interaction did not influence problematic SVA use directly, but exhibited an indirect relationship with problematic SVA use through flow. This finding further supported the appropriateness of the S-O-R framework in explicating problematic SVA use. Low-cost interaction is a behavior-related affordance that highlights the possibility of using SVAs with the least effort (Zhao, 2021). Notably, the perception of the low-cost interaction affordance indicated a cost-benefit evaluation of using SVAs, which suggested that the user was in a state of self-consciousness and was less likely to engage in problematic SVA use. Nevertheless, low-cost interaction largely facilitated users to experience flow, which in turn made users indulged themselves in problematic SVA use. Consequently, affordances related to users' assessment of usage behaviors tend not to cause problematic use directly but induce problematic use through triggering users' positive experiences such as flow.

Fourth, the findings indicate that different types of affordances may play different roles in explaining problematic SVA use. Within the techno-psychological approach, behavior-related affordances such as low-cost interaction serves as the most powerful predictor of problematic SVA use through flow, followed by form-related affordances such as multimodality. In comparison, content-related affordances such as recommendation algorithm may not be a consistent predictor, given its varying impacts on flow and problematic SVA use at different usage stages.

Fifth, although flow is often considered an optimal experience that helps individuals regulate their emotions and improve their wellbeing (Chen et al., 2022b), the role of flow in inducing problematic SVA use suggests its downside, which has been understudied in prior research. This reminds us to revisit flow in relation to mindfulness, a state of paying full attention to the present (Bishop et al., 2004; Regan et al., 2020; Liu et al., 2022a) and keeping a non-judgmental awareness of one's internal experiences (Baer et al., 2006; Kabat-Zinn, 2015). Overall, there are two approaches to understand flow and mindfulness (Chiou et al., 2022). One approach considers flow a branch of mindfulness because both flow and mindfulness focus on one's awareness of the present moment. This approach has been supported in studies that examined how mindfulness helped individuals stay focused and maintain awareness of the present in various settings (Chen et al., 2019, 2022a,b; Liu et al., 2022b). In contrast, another approach argues that flow and mindfulness are different from each other: mindfulness emphasizes one's self-awareness of his or her internal states, whereas an individual's self-awareness is largely weakened in the state of flow, due to an intense and focused concentration on the present (Reid, 2011). We take the second approach to view flow and mindfulness for two reasons. First, seeing flow as a state of losing reflective self-consciousness helps explain why users who experience flow tend to get addicted to using SVAs. Besides, arousing one's inner observer as suggested by mindfulness helps prevent users from engaging in flow-induced problematic SVA use (Lan et al., 2018; Regan et al., 2020; Arpaci, 2021; Lakshmi, 2021).

Last but not least, our proposed model not only corroborates the S-O-R framework but also enriches it in the digital environment. Furthermore, compared with prior research that used the S-O-R framework to examine how short-form videos' technological characteristics might facilitate users to develop SVA addiction (Tian et al., 2022), our study specifies and refines the technological affordances of SVAs and highlights the role of flow between these affordances and problematic SVA use. These findings suggest that the S-O-R framework and the techno-psychological approach are consistent with each other in explicating problematic use of SVAs and other digital applications.

### Practical implications

The findings have several implications for optimizing SVAs' affordances to properly manage problematic SVA use. Although the findings of our study did not support that recommendation algorithm was a technological affordance that caused problematic SVA use, frequent exposure to a narrowing array of contents had few benefits on users' psychological wellbeing. Thus, we first advise SVA platforms to improve the algorithms to recommend more diverse contents to users. For example, algorithms should draw user profiles from multiple dimensions, including users' interest, identity, behaviors, etc. Besides, updating user profiles in real time and randomly dividing users into several small groups both help algorithms push more diversified contents (Zhao, 2021). Meanwhile, alerts that remind users to pause use should be incorporated in the algorithm to prevent users from excessive use.

Second, given that multimodality had a direct and indirect link with problematic SVA use, SVA platforms could keep the commonly used modalities while lower the speed of updating various special effects. This measure has two advantages. On the one hand, keeping the essential modalities may prevent users from spending too much time on watching or creating short-form videos, thereby weakening their flow experience and lowering the tendency of engaging in problematic use. On the other hand, despite a decrease in the updates in special effects, the essential modalities provide users with the pleasure of using SVAs.

Third, the indirect association between low-cost interaction and problematic SVA use *via* flow suggests that SVA platforms should set some interruptive notifications. For instance, a notification reminding users to select certain pictures or characters could be set. Additionally, short quizzes could be another form of notification. These interruptive notifications would pop up if users continuously spent much time on SVAs (e.g., half an hour or longer). Through interruptions, notifications significantly increase the cost of interacting with SVAs and make temporal dissociation—an important element of flow—impossible, thus lowering the likelihood of performing problematic use.

On the part of users, we offer several suggestions to prevent them from developing problematic use behaviors. First of all, although flow is a psychological state that tends to induce problematic SVA use, mindfulness has the potential to intervene with the problematic use (Lan et al., 2018; Regan et al., 2020; Arpaci, 2021; Lakshmi, 2021). Thus, programs that cultivate users' mindfulness are recommended. Besides, users are advised to improve their digital literacy, namely, raising their awareness of the affordances of SVAs and improving their ability to properly use SVAs to fulfill desirable purposes. Lastly, given that duration of use is a predictor of problematic SVA use, users should develop a good habit of smartphone use, such as setting a time limit for using SVAs and other mobile applications.

### Limitations and future research

This study has several limitations. First of all, our survey data was cross-sectional. Therefore, the results demonstrated associations between the examined variables instead of causal relationships. To test the causal relationships depicted in the proposed model, future research is advised to use longitudinal surveys. For instance, researchers could measure participants' perceptions about recommendation algorithm, multimodality, and low-cost interaction in the first-wave survey. In the second-wave survey, flow and problematic SVA use could be measured.

Second, considering the nature of problematic SVA use, social desirability bias could exist in the measurement of this variable. Participants might answer the questions in a manner that will be viewed favorably by others (Krumpal, 2013). To overcome this bias, future research is advised to use objective data to measure problematic SVA use.

Third, we measured three technological affordances of SVAs, but we did not measure participants' psychological traits. Future research could take psychological traits into account and compare the relative impacts of affordances and psychological traits on flow. This links the techno-psychological approach to the more conventional psychopathological approach and may stimulate a conversation between the two.

Fourth, although stages of use might be a possible explanation for the negative association between recommendation algorithm and problematic SVA use, it is an inference with limited empirical evidence. Thus, researchers may interview managers of SVA platforms or invite technicians specialized in algorithms to participate in a focus-group discussion. Through these qualitative methods, the negative association between recommendation algorithm and problematic SVA use can be empirically explained.

Lastly, given that flow and mindfulness are closely related yet different to each other, future research could include mindfulness as a moderator in the mediating model proposed in the current study. Through survey or intervention studies, researchers may test whether mindfulness moderates the paths from technological affordances and flow and the path from flow and problematic SVA use. Findings from such a moderated mediation model could provide useful insights to develop intervention programs of problematic SVA use.

## Conclusion

This study proposes a mediation model to explore how three technological affordances of SVAs induce problematic SVA use through flow. Recommendation algorithm directly and negatively predicts problematic SVA use. Multimodality is directly and positively linked to problematic SVA use, while has an indirect link with the outcome variable *via* flow. Low-cost interaction is found to be indirectly related to problematic SVA use through flow. The results demonstrate the difference in the associations between each affordance and problematic SVA use. Moreover, our findings suggest a techno-psychological approach to examining problematic use of SVAs and other smartphone applications.

The psychopathological approach assumes that individuals with predisposed psychopathologies are highly vulnerable to problematic use behaviors (Davis, 2001; Elhai and Contractor, 2018; Elhai et al., 2020). To challenge such an assumption, the compensatory use approach proposes that problematic use represents an unintended consequence resulted from people's active use of digital tools to compensate for the undesirable life situations (Kardefelt-Winther, 2014; Elhai et al., 2017; Wegmann and Brand, 2019). Following this line of research, the techno-psychological approach extends the compensatory use approach through introducing technological affordances as antecedents of users' psychological experiences and subsequent behaviors. Against the backdrop of the digital age, the techno-psychological approach offers us insights into understanding how digital technologies afford the development of problematic use behaviors, and provides us with some implications for optimizing digital applications to effectively manage the problematic use of various applications.

## Data availability statement

The raw data supporting the conclusions of this article will be made available by the authors, without undue reservation.

## Ethics statement

The studies involving human participants were reviewed and approved by the Institutional Review Board of the Faculty of Social Sciences, Zhejiang University. The patients/participants provided their written informed consent to participate in this study.

## Author contributions

QH acquired the funding, designed the study, analyzed the data, and wrote the main body of the manuscript. MH participated in the study design, cleaned the data, wrote the Method and Result sections, and made the tables and figures. NZ acquired the funding, participated in the study design, and reviewed and revised the manuscript. All authors contributed to the article and approved the submitted version.

## Funding

This study was supported by the Fundamental Research Funds for the Central Universities from Zhejiang University.

## Conflict of interest

The authors declare that the research was conducted in the absence of any commercial or financial relationships that could be construed as a potential conflict of interest.

## Publisher's note

All claims expressed in this article are solely those of the authors and do not necessarily represent those of their affiliated organizations, or those of the publisher, the editors and the reviewers. Any product that may be evaluated in this article, or claim that may be made by its manufacturer, is not guaranteed or endorsed by the publisher.

## Footnotes

^1^Non-significant p-value for Chi-square, CFI > 0.95, TLI > 0.95, GFI > 0.95, RMSEA < 0.06, SRMR < 0.06.
